# Recovery from blindness following accidental quinine overdose

**DOI:** 10.1136/practneurol-2017-001610

**Published:** 2017-08-04

**Authors:** Hildegard Nikki Hall, Andrew J Tatham

**Affiliations:** 1 Princess Alexandra Eye Pavilion, Edinburgh, UK; 2 MRC Human Genetics Unit, MRC Institute for Genetics and Molecular Medicine, University of Edinburgh, Edinburgh, UK

**Keywords:** quinine, visual loss, cinchonism, neurotoxicology, ophthalmology

## Case

A 46-year-old woman presented with sudden loss of vision in both eyes, apparent since waking. She also complained of partial hearing loss. The previous evening she had experienced difficulty sleeping due to (long-standing) restless legs and had self-medicated with quinine, taking an accidental overdose of 1.8 g.

On admission, she was alert but unable to perceive light in either eye, and had fixed, dilated pupils. Other than mild bilateral sensorineural hearing loss, the remainder of the neurological examination was normal. She was haemodynamically stable, but her ECG showed QT interval prolongation at 501 ms (normal 350–440); she therefore received intravenous sodium bicarbonate for suspected myocardial toxicity.

Ophthalmological review that day confirmed a visual acuity of no light perception and a bilateral afferent pupillary defect. Fundal examination suggested mild retinal oedema. Given the profound visual loss, we prescribed a 4-week course of oral nimodipine 60 mg 4-hourly, based on limited evidence from case series.^1^

## Progress

**Figure 1:**
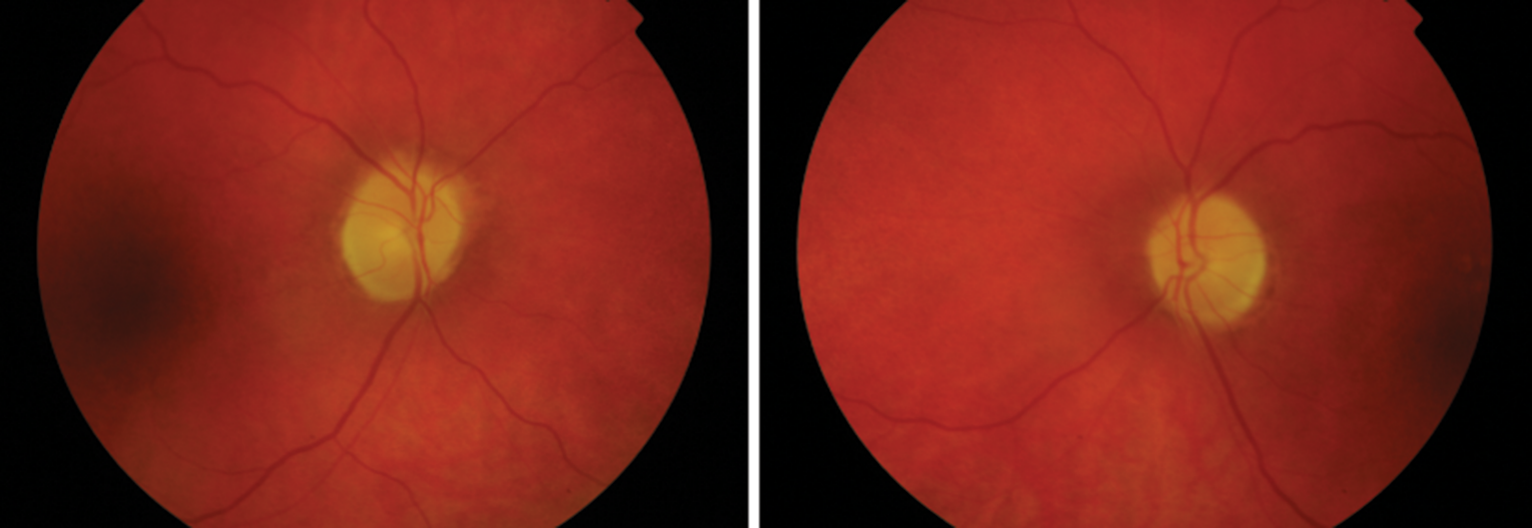
Fundus photographs showing pale discs and retinal vessel attenuation. The patient’s fundus appearance was unremarkable in the early stages; the changes seen here were seen from 1 month postoverdose and are secondary to the widespread quinine-induced retinal toxicity. These photographs were taken at 6 months, at which point there had been some recovery of the visual function but no further change to the funduscopic appearance.

**Figure 2:**
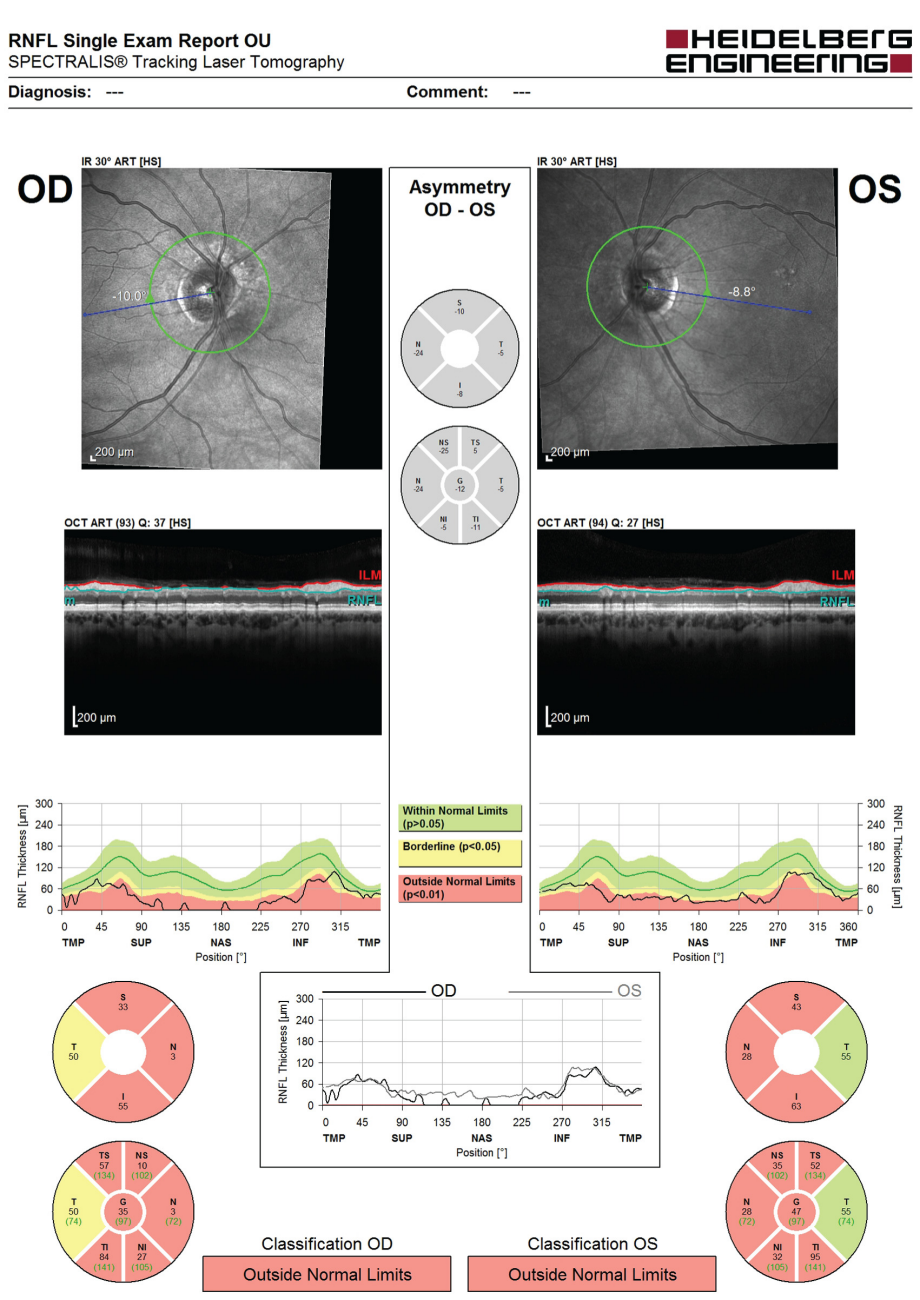
Optical coherence tomography showing thinning of the retinal nerve fibre layer (RNFL) 1 month postquinine overdose.  OU, oculus uterque (both eyes); OD, oculus dexter (right eye); OS, oculus sinister (left eye).

**Figure 3:**
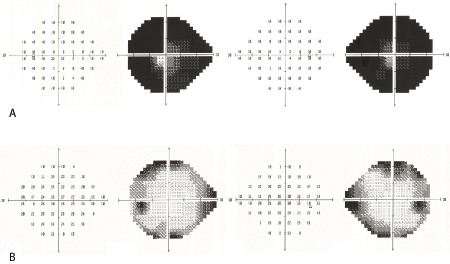
Humphrey perimetry (SITA-Standard 24–2): (A) 1 month postquinine overdose, showing marked constriction of the visual field; (B) 6 months postquinine overdose, showing clear improvement of the visual field defects.

Two days postoverdose, her vision started to return such that she could perceive light. Slit lamp fundus examination was normal at this point. Her sight gradually improved. By 1 month, visual acuity was 6/9 bilaterally, although subjectively her vision was akin to ‘being under water’. Colour vision was profoundly reduced: she could discern the Ishihara control plate only. Her pupils remained persistently dilated with sluggish direct reactions to light. Funduscopy now showed optic disc pallor and retinal vessel attenuation bilaterally (figure 1), with thinning of the nasal retinal nerve fibre layer on optical coherence tomography (figure 2). Visual fields were severely constricted (figure 3A) such that she remained functionally limited. Electrodiagnostic testing at 3 months was consistent with quinine overdose, with reduced electroretinogram amplitude and abnormal flicker response.

Over 6 months, her visual fields expanded significantly (figure 3B), reflecting partial recovery of retinal function following quinine toxicity.

## Discussion

Acute quinine toxicity produces a well-recognised constellation of clinical effects, known as cinchonism. This case demonstrated visual and hearing loss as well as signs of cardiac instability. In addition, cinchonism may comprise tinnitus, dizziness, headache, seizures and vomiting. Quinine is believed to be directly toxic to the inner retina^2^ and, while the natural history is of some recovery over days to weeks, residual visual field constriction is common and the vision may remain poor.

There is no high-quality evidence for any treatment for quinine-related blindness. However, due to the arteriolar constriction seen in some, there have been several case reports of treatment to alleviate vasospasm with agents such as nimodipine.^1^ While the retinal vasculature was unremarkable at initial assessment, we prescribed nimodipine based on the very poor level of vision and good safety profile of the medication.

Due to the narrow therapeutic range of quinine, visual effects can also rarely occur at normal doses. Furthermore, as quinine is a commonly prescribed medication, it is not always readily identified as the culprit. It should be suspected particularly in the presence of fixed, dilated pupils and other features of cinchonism.
